# Morphology and multigene phylogeny reveal two new species of *Micropsalliota* (*Agaricaceae*, *Basidiomycota*) from Dhofar region, southern Oman

**DOI:** 10.3897/mycokeys.137.192271

**Published:** 2026-07-28

**Authors:** Hamed Al-Nadabi, Shah Hussain, Ahmed Al Sidairi, Khalifa Al-Zeidi, Ahmed Al-Raeesi, Rahil Al-Badi, Lujain Al-Rashdi, Jumana Al-Jashmi, Rethinasamy Velazhahan, Abdullah M. Al-Sadi

**Affiliations:** 1 Center for Environmental Studies and Research (CESAR), Sultan Qaboos University, P.O. Box 34, Al-Khoud 123, Muscat, Oman College of Agriculture, University of Al Dhaid Sharjah United Arab Emirates; 2 Department of Plant Sciences, College of Agricultural and Marine Sciences, Sultan Qaboos University, Muscat, Oman Center for Environmental Studies and Research (CESAR), Sultan Qaboos University Muscat Oman; 3 College of Agriculture, University of Al Dhaid, Sharjah P.O. Box 27272, United Arab Emirates Department of Plant Sciences, College of Agricultural and Marine Sciences, Sultan Qaboos University Muscat Oman; 4 Muscat University, P.O. Box 550, PC 130, Al Ghubra North, Muscat, Oman Muscat University Muscat Oman

**Keywords:** Agarics, clade, megaspora, taxonomy, termite mounds

## Abstract

*Micropsalliota* is a genus of small to relatively medium-sized saprotrophic mushrooms, with fibrillose-squamulose pileus surface, ellipsoid to amygdaliform or cymbiform basidiospores, usually with capitate cheilocystidia, and encrusted pileus hyphae. In this study, two new species, namely *Micropsalliota latiumbonata* and *M. striata* are described from Oman, based on morphology and multigene (ITS-28S-*rpb2*-*tef1-α*) phylogenetic analysis. *Micropsalliota latiumbonata* is characterized by a broadly umbonate pileus disc with fibrillose-squamulose surface, fibrils dark-brown and more or less recurved, cheilocystidia capitate with olivaceous capitulum, broadly fusiform to amygdaliform basidiospores measuring 6.0–7.0 × 4.0–4.5 µm, and occurring gregariously around termite mounds. *Micropsalliota striata* is another small-sized species with an acute papillate pileus disc with appressed fibrils, striate-appendiculate pileus margins, utriform cheilocystidia without a capitate apex, clavate to subglobose pileocystidia, and comparatively larger basidiospores (6.5–7.5 × 3.7–4.1 µm). Both the new species belong to Clade megaspora. A detailed description, photographs of basidiomata, illustrations of anatomical characteristics, a multigene phylogenetic tree, and morphological comparison with similar species are provided. With the description of these two species, the number of known species in *Micropsalliota* rises to 108.

## Introduction

The genus *Micropsalliota* Höhn. is a group of small, slender mushrooms, forming the basal lineage in the subfamily *Leucocoprinoideae* R.L. Zhao & J.X. Li within *Agaricaceae* Chevall. (Li et al. 2025). Morphologically, *Micropsalliota* has the following combination of characters, including agaricoid, small to relatively medium-sized basidiomata (except *M. geesterani* (Bas & Heinem.) R.L.Zhao & L.A.Parra with large basidiomata), fibrillose to squamulose pilei with fibrils/squamules often brownish, reddish or whitish in color, usually with umbonate pileus disc, annulus membranous (if present), brownish spore print, basidiospores ellipsoid to amygdaliform usually with apical thickening, cheilocystidia mostly capitate or subcapitate, and more or less with incrusted pileus hyphae (Al-Kharousi et al. 2022; Li et al. 2025).

In recent years, the taxonomic work on *Micropsalliota* has been accelerated with the introduction of many new species on the basis of morphology and DNA sequence data (Zhao et al. 2010; Wei et al. 2015; Chen et al. 2016; He et al. 2020; Li et al. 2021; Patil et al. 2022, 2025; Yan et al. 2022; Gao et al. 2024; Li et al. 2025; Wang et al. 2026). In a systematic study of *Agaricaceae* s.l. using genomic data, it was found that *Micropsalliota* forms a basal lineage with genera such as *Leucocoprinus*, *Leucoagaricus*, and *Macrolepiota*, collectively forming the subfamily *Leucocoprinoideae* (Li et al. 2025).

Currently, more than 100 *Micropsalliota* species have been described worldwide, distributed in tropical and subtropical regions of Africa (Heinemann 1983; Cao et al. 2025; Liu et al. 2025), Americas (Heinemann 1989; Guzmán-Dávalos and Heinemann 1994) and Asia (Zhao et al. 2010; Wei et al. 2015; Chen et al. 2016; He et al. 2020; Li et al. 2021; Patil et al. 2022, 2025; Yan et al. 2022; Gao et al. 2024; Li et al. 2025; Wang et al. 2026). Previously, only *Micropsalliota ventricocystidiata* Al-Sadi & S. Hussain has been reported from Oman (Al-Kharousi et al. 2022).

The southern part of Oman (Dhofar Governorate) experiences monsoon rains in July to September each year, which influence the growth and development of various kinds of mushrooms (Hussain et al. 2022, 2024a, 2024b, 2025a, 2025b). The area of Dhofar which is under the influence of monsoon rains, has a large population of a termite species (*Macrotermes subhyalinus*), making huge termite mounds (AlShamakhi et al. 2024). It was observed that these termite mounds provide a favorable habitat for the growth of various mushrooms (Hussain et al. 2024b, 2024c, 2026).

In this study, several mushroom specimens were collected during the 2025 monsoon season. Morphological characterization of basidiomata and multigene phylogenetic analyses showed that these collections represent two new species in the genus *Micropsalliota*, which are described here.

## Materials and methods

### Specimen collections and morphology

Basidiomata were collected during the rainy season, in August-September 2025, from Ain Athum, Ashwur and Qashrub areas, near Salalah, Dhofar Governorate, the southern region of the Sultanate of Oman. The basidiomata were photographed in their natural habitat, tagged with collection information (Rathnayaka et al. 2025), and macromorphological characters, including basidiomata size, shape, and color (according to Munsell Soil Color Charts; Munsell 1975) were recorded. The specimens were brought to the field station, where the fruiting bodies were dried at 45 °C in a fruit dehydrator (Semikorn Electronic Technology Co., Ltd., Guangdong, China). After drying, the samples were sealed in zipper bags and stored at −80 °C for two weeks to eliminate any insect eggs or larvae. The studied materials were deposited in the Fungal Collections at the Center for Environmental Studies and Research (CESAR), Sultan Qaboos University, Oman.

A thin hand-cut section from pileus covering, lamellae, and stipe was prepared for microscopic observation, using 5% aqueous KOH (w/v) and stained in 1% Congo red (w/v) for better contrast. Microscopic features, including the size, shape, color, surface features of basidiospores, basidia, cheilo-, pleuro-, and caulocystidia, and pileipellis were observed under a light microscope (ECLIPSE N*i*-U, Nikon Co., Ltd., Japan). Basidiospores’ notations are presented as: [*n/o/p*] where *n* is the number of basidiospores measured, from *o* number of basidiomata, and from *p* collections; the dimensions (a)b–c(d), where b–c includes at least 90% of measurements and ‘a’ and ‘d’ are extreme values. Q is the length/width ratio for individual spores; av. Q is the mean Q. For morphological description, Bas et al. (2011) was followed.

### DNA extraction, amplification, and sequencing

DNA was extracted from dried specimens using Tissue DNA Preparation – Column Kit (Jena Bioscience GmbH, Jena, Germany). Three DNA regions were amplified, including internal transcribed spacer (ITS) and D1/D2 domains of the large subunit of nuclear ribosomal DNA (28S), and a segment of a protein-coding gene, the translation elongation factor 1 alpha (*tef1-α*). The primers were ITS5/ITS4 for ITS (Gardes and Bruns 1993; White et al. 1990), LR0R/LR5 for 28S (Vilgalys and Hester 1990), and EF1-983F/EF1-1567R for *tef1-*α (Rehner and Buckley 2005), respectively. Conditions for polymerase chain reactions (PCR) were set as: an initial denaturation at 94 °C for 3 min, followed by 35 cycles of denaturation at 94 °C for 30 s, annealing at the gene-specific temperature and duration (ITS: 56 °C for 40 s; 28S: 52 °C for 45 s; *tef1-*α: 60 °C for 46 s), extension at 72 °C for 50 s, and a final extension at 72 °C for 10 min, followed by a 4 °C soak. PCR reactions were performed with PuReTaq Ready-To-Go PCR beads (GE Healthcare UK Limited, Buckinghamshire, UK). These reagents were added to each PCR bead: 1.0 µL of each primer (10 µmol/L), 2 µL of DNA template, and 21 µL nuclease-free water. The amplified regions were purified and sequenced with same primers at Macrogen Inc. © (Seoul, Republic of Korea).

### Sequence alignment and phylogenetic analyses

The newly generated sequences of both forward and reverse primers of ITS, 28S, and *tef1-α* were cleaned to consensus sequences using BioEdit v. 7.0.9 (Hall 1999). In BLAST searches, the ITS sequences of the proposed new species *Micropsalliota latiumbonata* showed the highest similarity of 96.56% with an undescribed species from Oman *M.* sp. DRB-23-013 (PV088726) and 95.01% with *M. appendiculata* LE F-315913 (OR161109, Vietnam), while 2^nd^ new species *M. striata* has 97.50% similarity with the undescribed species *M.* sp. DRB-23-013 (PV088726, Oman). A combined dataset of ITS-28S-*rpb2*-*tef1-α* was constructed (Gao et al. 2024; Yan et al. 2025). The final dataset comprised 112 specimens, including 109 *Micropsalliota* specimens and three outgroup taxa (*Agaricus arabiensis* SQUH-SNT007, *Coniolepiota spongodes* PNG012/MFLU 09-0012, and *Hymenagaricus wadijarzeezicus* JHN-22-019). These taxa were used as outgroups because they belong to the subfamily *Agaricoideae* R.L. Zhao & J.X. Li (*Agaricaceae*), a lineage phylogenetically distinct from the ingroup taxa, making them suitable for rooting the phylogenetic tree (Li et al. 2025). The dataset was based on the sequences employed in the recent phylogenetic studies of *Micropsalliota* (Yan et al. 2022; Gao et al. 2024; Yan et al. 2025). The dataset was aligned using MAFFT v.7 (Katoh et al. 2019), and adjusted in BioEdit v.7.0.9.0 (Hall 1999) and finally in ClustlX v. 2.1 (Larkin et al. 2007). Details of the sequences used in this study are given in Table 1.

**Table 1.** Sequence information used in the phylogenetic analyses of *Micropsalliota*, bold are the newly generated sequences, (–) means data unavailable.

**Taxa**	**Vouchers**	**Country**	**ITS**	**28S**	***rpb2***	***tef1-α***	**Reference**
*Micropsalliota alba*	–	India	EF069420	–	–	–	Gao et al. 2024.
*M. albella*	LE2016123	Thailand	MN294514	MN294516	–	–	He et al. 2020.
*M. albofelina*	LE312536	Vietnam	OK257212	OK257209	–	–	Crous et al. 2021.
*M. albofelina*	HKAS70329	China	OR799877	OR799922	OR962218	OR962180	Gao et al. 2024.
*M. albosericea*	SFSU-zrl-3049	Thailand	HM436644	–	–	–	Zhao et al. 2010.
*M. allantoidea*	SFSU-zrl-2038	Thailand	HM436648	HM436597	–	–	Zhao et al. 2010.
*M. appendiculata*	HKAS131127	China	OR799912	OR799956	OR962247	OR962204	Gao et al. 2024.
*M. appendiculata*	LE F-315913	Vietnam	OR161109	OR161104	–	–	Ivanova et al. 2023.
*M. arginophaea*	SFSU-zrl-3110	Thailand	HM436617	HM436577	–	–	Zhao et al. 2010.
*M. arginophaea*	HKAS60309	China	OR799878	OR799923	OR962219	OR962208	Gao et al. 2024.
*M. bifida*	SFSU-zrl-3067	Thailand	HM436640	HM436591	–	–	Zhao et al. 2010.
*M. bifida*	HFJAU2998	China	OM650272	OM650252	OM669858	–	Yan et al. 2022.
*M. bispora*	HFJAU3833	China	PQ345346	PQ345351	PQ358515	PQ358519	Yan et al. 2025
*M. bispora*	HFJAU4253	China	PQ345347	PQ345352	PQ358516	PQ358520	Yan et al. 2025
*M. brunneosquamata*	LD201236	Thailand	KP316210	–	–	–	Chen et al. 2016.
*M. cortinata*	SFSU-zrl-2129	Thailand	HM436630	HM436593	–	–	Zhao et al. 2010.
*M. cortinata*	HKAS92221	China	OR799879	OR799924	OR962220	OR962183	Gao et al. 2024.
*M. delicatula*	HKAS54332	China	OR799880	OR799925	OR962221	OR962209	Gao et al. 2024.
*M. delicatula*	ZRL2015234	China	MT671229	–	–	–	Li et al. 2021.
*M. dentatomarginata*	GX20170202	China	MT671228	MT671242	–	–	Li et al. 2021.
*M. digitatocystis*	HKAS123832	China	OR799883	OR799928	OR962224	OR962185	Gao et al. 2024.
*M. digitatocystis*	ZRL20180564	China	MT671239	MT671250	–	–	Li et al. 2021.
*M. ferruginea*	HKAS131130	China	OR799885	OR799930	OR962226	OR962182	Gao et al. 2024.
*M. ferruginea*	HKAS70562	China	OR799884	OR799929	OR962225	OR962181	Gao et al. 2024.
*M. fimbriata*	HKAS60241	China	OR799886	OR799931	OR962227	OR962198	Gao et al. 2024.
*M. fimbriata*	HKAS60261	China	OR799887	OR799932	–	OR962199	Gao et al. 2024.
*M. furfuracea*	SFSU-zrl-3006	Thailand	HM436621	HM436603	–	–	Zhao et al. 2010.
*M. furfuracea*	HKAS60229	China	OR799889	OR799934	OR962229	OR962201	Gao et al. 2024.
*M. geesterani*	LAPAG520	Thailand	KM923965	KM923966	–	–	Parra et al. 2016.
*M. geesterani*	E.C. Vellinga 2263(L)	Netherlands	AF482857	AF482888	–	–	Parra et al. 2016.
*M. gigaspora*	HKAS131118	China	OR799890	OR799935	OR962230	–	Gao et al. 2024.
*M. gigaspora*	HKAS131119	China	OR799891	OR799936	–	–	Gao et al. 2024.
*M. globocystis*	HKAS131120	China	OR799892	OR799937	–	OR962186	Gao et al. 2024.
*M. globocystis*	HFJAU1518	China	OM650277	OM650255	OM669852	–	Yan et al. 2022.
*M. globocystis*	HKAS131133	China	OR799895	OR799940	OR962234	OR962197	Gao et al. 2024.
*M. globocystis*	HKAS92202	China	OR799894	OR799939	OR962233	OR962196	Gao et al. 2024.
*M. globocystis*	VDW1278	South Africa	MT304640	–	–	–	Yan et al. 2022.
*M. globocystis*	SFSU-zrl-2049	Thailand	HM436635	–	–	–	Zhao et al. 2010.
*M. globocystis*	HFJAU2709	China	OM650278	OM650262	OM669856	–	Yan et al. 2022.
*M. globovelveta*	HFJAU3842	China	PV277734	PV269809	PV289604	PV289605	Liu et al. 2025
*M. gracilis*	SFSU-zrl-2041	Thailand	HM436647	HM436583	–	–	Zhao et al. 2010.
*M. gracilis*	HNL503432	Laos	MW192914	–	–	–	Yan et al. 2022.
*M. inflata*	LE F-315912	Vietnam	OR161110	OR161106	–	–	Ivanova et al. 2023.
*M. jiangxiensis*	THJ20018	China	ON117420	ON117438	–	–	Ji and He 2023.
*M. jiangxiensis*	THJ20019A	China	ON117421	ON117439	–	–	Ji and He 2023.
***M. latiumbonata***	**QRB-14-25**	**Oman**	**PZ127411**	**PZ127514**	–	**PZ390627**	**This study**
***M. latiumbonata***	**ASH-005-25**	**Oman**	**PZ127410**	**PZ127513**	–	**PZ390625**	**This study**
***M. latiumbonata***	**ASH-006-25**	**Oman**	**PZ127412**	**PZ127515**	–	**PZ390626**	**This study**
***M. latiumbonata***	**ASH-007-25**	**Oman**	**PZ127413**	**PZ127516**	–	**PZ386150**	**This study**
***M. latiumbonata***	**ASH-009-25**	**Oman**	**PZ127414**	**PZ127517**	–	**PZ176529**	**This study**
*M. lateritia*	SFSU-zrl-2073	Thailand	HM436631	–	–	–	Zhao et al. 2010.
*M. lateritia*	HKAS131124	China	OR799896	OR799941	OR962235	OR962202	Gao et al. 2024.
*M. longicystis*	HKAS131121	China	OR799897	OR799942	OR962257	–	Gao et al. 2024.
*M. longicystis*	HKAS131126	China	OR799898	OR799943	OR962258	–	Gao et al. 2024.
*M. megarubescens*	SFSU-zrl-2086	Thailand	HM436620	–	–	–	Zhao et al. 2010.
*M. megarubescens*	HKAS60253	China	OR799900	OR799945	OR962237	OR962189	Gao et al. 2024.
*M. megaspora*	SFSU-zrl-3068	Thailand	HM436624	–	–	–	Zhao et al. 2010.
*M. megaspora*	HFJAU1255	China	OM650282	OM650258	OM669876	–	Yan et al. 2022.
*M. minor*	HFJAU2796	China	OM650294	OM650266	OM669865	–	Yan et al. 2022.
*M. nana*	HKAS114619	China	OR799901	OR799946	OR962238	OR962216	Gao et al. 2024.
*M. nana*	HKAS115226	China	OR799902	OR799947	–	OR962217	Gao et al. 2024.
*M. ovalispora*	HFJAU2010	China	OM650295	OM650269	OM669866	–	Yan et al. 2022.
*M. ovalispora*	HFJAU3179	China	OM650296	–	OM669867	–	Yan et al. 2022.
*M. pakistanica*	LAH38139	Pakistan	PP357040	PP383879	–	–	Maula et al. 2025
*M. pakistanica*	LAH38140	Pakistan	PP357041	PP383880	–	–	Maula et al. 2025
*M. pileocystidiata*	AMH9975	India	MG917970	–	–	–	Patil et al. 2022.
*M. pileocystidiata*	MMH1114	India	MZ598496	–	–	–	Patil et al. 2022.
*M. pleurocystidiata*	SFSU-zrl-2023	Thailand	HM436636	–	–	–	Zhao et al. 2010.
*M. pseudoarginea*	HKAS131125	China	OR799903	OR799948	OR962239	OR962212	Gao et al. 2024.
*M. pseudoarginea*	HFJAU2122	China	OM650284	OM650260	OM669861	–	Yan et al. 2022.
*M. pseudodelicatula*	HKAS131129	China	OR799905	OR799950	OR962241	OR962213	Gao et al. 2024.
*M. pseudodelicatula*	HFJAU2228	China	OM650288	OM650264	OM669863	–	Yan et al. 2022.
*M. pseudoglobocystis*	HKAS87127	China	OR799908	OR799953	OR962244	OR962190	Gao et al. 2024.
*M. pseudoglobocystis*	ZRL2013321	China	KM889913	–	–	–	Wei et al. 2015.
*M. purpureobrunneola*	LE2016124	Thailand	MN294513	MN294517	–	–	He et al. 2020.
*M. pusillissima*	SFSU-zrl-3047	Thailand	HM436645	HM436594	–	–	Zhao et al. 2010.
*M. repanda*	LAPAF8	Togo	KP739805	KP739804	–	–	Parra et al. 2016.
*M. rosea*	LAH38149	Pakistan	PP377825	PP378506	–	–	Maula et al. 2025
*M. rosea*	LAH38186	Pakistan	PQ623104	PQ623106	–	–	Maula et al. 2025
*M. roseipes*	HFJAU2494	China	OM650297	OM650270	OM669870	–	Yan et al. 2022.
*M. rubrobrunnescens*	SFSU-zrl-2120	Thailand	HM436628	HM436588	–	–	Zhao et al. 2010.
*M. rubrobrunnescens*	HKAS96929	China	OR799914	OR799958	OR962249	OR962206	Gao et al. 2024.
*M. rubrobrunnescens*	SFSU-zrl-2121	Thailand	HM436629	HM436589	–	–	Zhao et al. 2010.
*M. rufosquarrosa*	HFJAU1208	China	OM650291	OM650267	OM669868	–	Yan et al. 2022.
*M. rufosquarrosa*	HFJAU1236	China	OM650292	OM650268	OM669869	–	Yan et al. 2022.
*M. squarrosa*	HKA128713	China	OR799916	OR799960	OR962251	–	Gao et al. 2024.
*M. squarrosa*	HKAS128633	China	OR799915	OR799959	OR962250	–	Gao et al. 2024.
*M.* sp.	DRB-23-013	Oman	PV088726	PV093920	–	–	Unpublished
***M. striata***	**ATM-009-25**	**Oman**	**PZ127415**	**PZ127518**	–	**–**	**This study**
*M. subalba*	SFSU-zrl-2080	Thailand	HM436646	HM436596	–	–	Zhao et al. 2010.
*M. subalba*	HKAS105828	China	OR799917	OR799961	–	OR962211	Gao et al. 2024.
*M. subarginea*	SFSU-zrl-2052	Thailand	HM436612	HM436573	–	–	Zhao et al. 2010.
*M. subarginea*	SFSU-zrl-2092	Thailand	HM436611	HM436574	–	–	Zhao et al. 2010.
*M. subumbonata*	AMH 10512	India	PP837559	PQ576415	–	–	Patil et al. 2025.
*M. subumbonata*	MMH 2312	India	PQ577838	PQ580740	–	–	Patil et al. 2025.
*M. suricatoides*	LE F-348071	Vietnam	OR161111	OR161105	–	–	Ivanova et al. 2023.
*M. suricatoides*	LE F-348072	Vietnam	OR161112	–	–	–	Ivanova et al. 2023.
*M. tenuipes*	HFJAU1536	China	OM650289	–	–	–	Yan et al. 2022.
*M. tenuipes*	HFJAU3180	China	OM650290	OM650265	–	–	Yan et al. 2022.
*M. umbonata*	HKAS131131	China	OR799920	OR799964	OR962254	OR962194	Gao et al. 2024.
*M. umbonata*	HKAS131132	China	OR799921	OR799965	OR962255	OR962195	Gao et al. 2024.
*M. ventricocystidiata*	SQUH-ATR004	Oman	OM397373	OM630413	–	–	Al-Kharousi et al. 2022.
*M. ventricocystidiata*	SQUH-GOB002	Oman	OM397374	OM630414	–	–	Al-Kharousi et al. 2022.
***M. ventricocystidiata***	**JEB-001-25**	**Oman**	**PZ127409**	**PZ127512**	–	**PZ176528**	**This study**
*M. vulgaris*	HFJAU3350	China	PQ345344	PQ345349	PQ358513	PQ358517	Yan et al. 2025.
*M. vulgaris*	HFJAU5707	China	PQ345345	PQ345350	PQ358514	PQ358518	Yan et al. 2025.
*M. wuyishanensis*	HFJAU3048	China	OM650298	–	OM669878	–	Yan et al. 2022.
*M. xanthorubescens*	SFSU-zrl-3083	Thailand	HM436638	HM436598		–	Zhao et al. 2010.
*M. xanthorubescens*	SFSU-zrl-3096	Thailand	HM436637	HM436599		–	Yan et al. 2022.
*Agaricus arabiensis*	SQUH-SNT007	Oman	OM971855	OM971859		ON568585	Hussain et al. 2022.
*Hymenagaricus wadijarzeezicus*	JHN-22-019	Oman	OR612998	OR613021		OR729600	Hussain et al. 2024b.
*Coniolepiota spongodes*	PNG012/MFLU 09-0012	Thailand	HM488756	HM488774	HM488796	HM488883	Vellinga et al. 2011.

**Figure 1.**
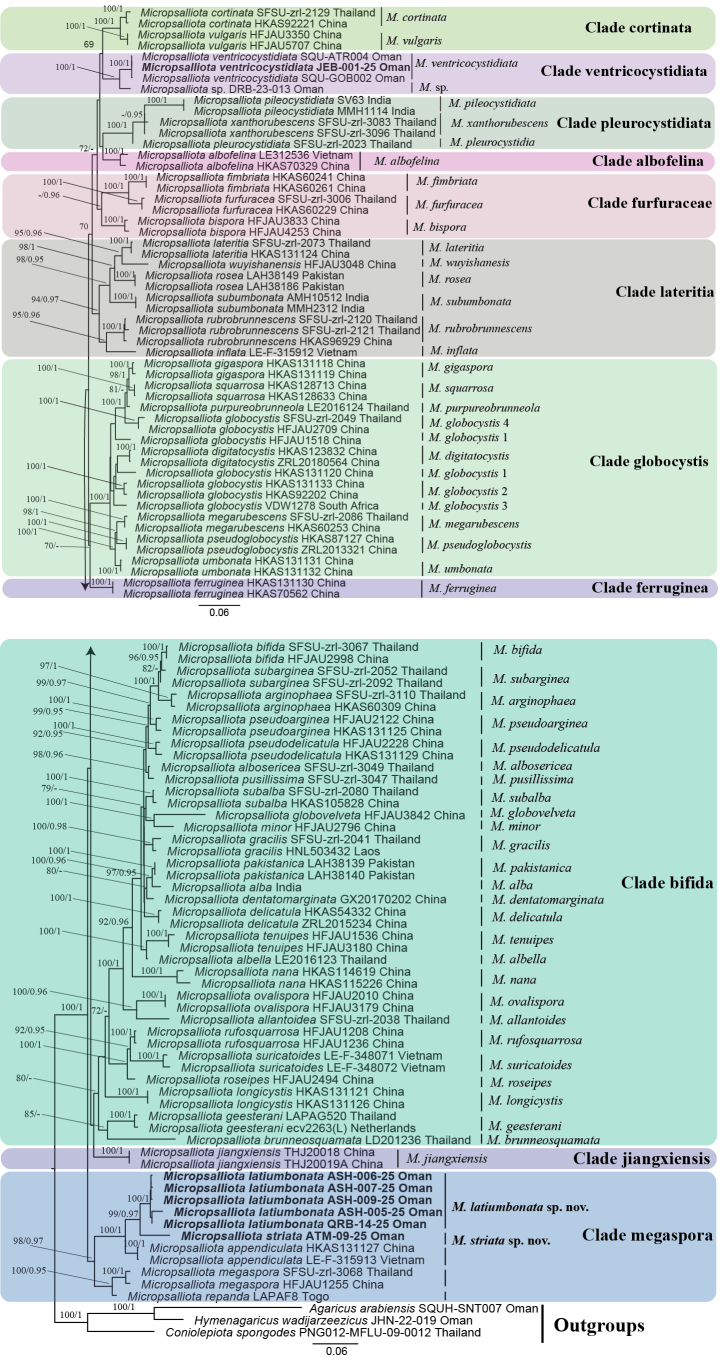
Multigene phylogeny of *Micropsalliota* generated by ML analyses, based on combine sequence data of ITS-28S-*rpb2*-*tef1-α*, with *Agaricus arabiensis*, *Coniolepiota spongodes*, and *Hymenagaricus wadijarzeezicus* as outgroup taxa. Bayesian posterior probabilities (PPs) and ML bootstrap percentage (BT) ≥ 0.95 and ≥70%, respectively, are shown above the nodes. Eleven clades were recovered, each branch represents species name, followed by voucher number, and country of origin, specimens studied during this work are in bold fonts, and both the new species are grouped in Clade megarspora.

Maximum likelihood (ML) and Bayesian Inference (BI) methods were used for phylogenetic analyses. The BI phylogeny was performed using MrBayes v. 3.2.6 (Ronquist et al. 2012), and the best-fit model (GTR+F+I+G4) was selected following ModelFinder (Kalyaanamoorthy et al. 2017). Four Markov chains were run each for one million generations and sampled every 100^th^ generations. Burn-in was determined by checking the likelihood trace plots in Tracer v. 1.7 (Rambaut et al. 2018) and subsequently discarded. Maximum likelihood (ML) analyses were tested with RAxML v. 8 (Stamatakis 2014) with 1000 bootstrap replicates. The best model (GTR+I+G) was chosen according to the corrected Akaike information criteria (AICc) (Lanfear et al. 2017). The BI posterior probabilities (PPs) and ML bootstrap percentages (BT) of ≥ 0.95 and ≥ 70%, respectively were considered significant and shown on phylogenetic tree (Fig. 1) in order of (BT/PPs). Tree files were visualized with FigTree v.1.4.2 (Rambaut 2012) followed by annotation in Adobe Illustrator CC2019.

## Results

### Phylogenetic analyses

The final aligned multigene dataset consisted of 112 specimens with 3310 characters long, including 1875 constant sites, 980 informative sites, and 455 uninformative sites. These 112 specimens represent 55 named species, and one unnamed species of the genus *Micropsalliota*, along with the three outgroup taxa. Both ML and BI methods generated phylogenies with similar topologies. The ML tree is shown (Fig. 1) with values of both maximum likelihood BT percentage and Bayesian PPs. The presented tree is almost similar to previous phylogenies of *Micropsalliota* (Gao et al. 2024; Yan et al. 2025), where 109 specimens of the genus grouped in 11 clades, each with strong to excellent phylogenetic support. The representative new species, *Micropsalliota latiumbonata* and *M. striata*, are grouped in the Clade megaspora with strong phylogenetic support (98%/0.97).

### Taxonomy

#### Micropsalliota latiumbonata

Taxon classification

Fungi

AgaricalesAgaricaceae

S. Hussain, Al-Nadabi & Al‐Sadi sp. nov.

63BE31AA-8667-513D-A072-E6C7143605B3

862914

Figs 2, 3

##### Etymology.

The specific epithet ‘latiumbonata’ refers to the broadly umbonate pileus disc of the new species.

**Figure 2.**
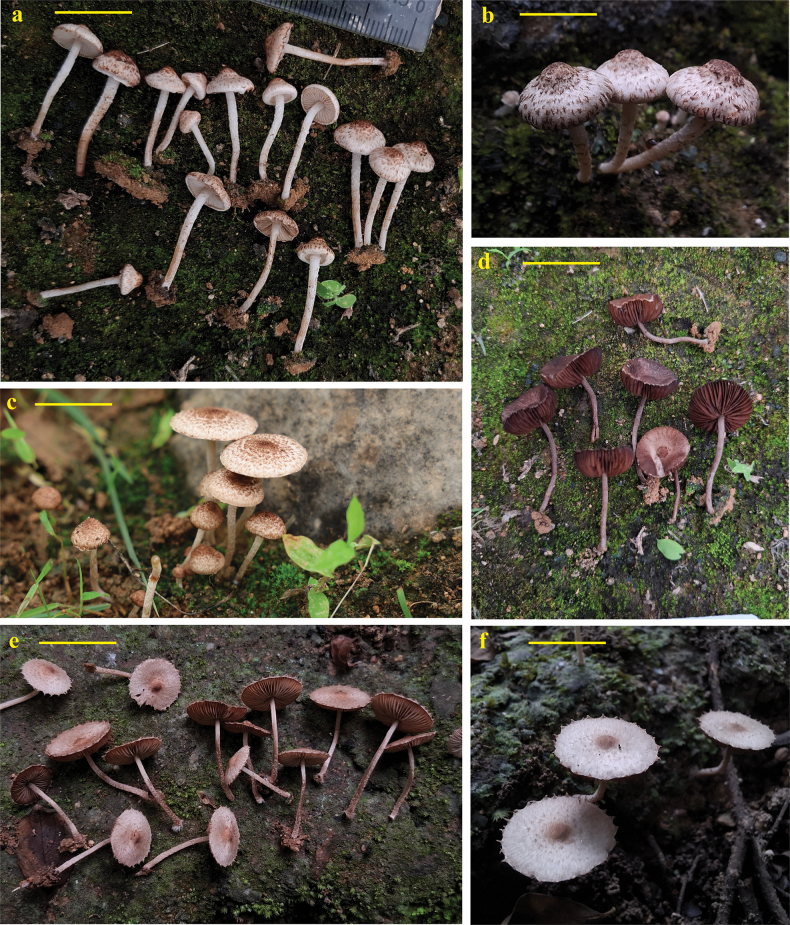
Basidiomata of the new species of *Micropsalliota*. **a–d**. *Micropsalliota latiumbonata*. **a, b**. (CESAR-ASH-007-25, holotype); **c**. ASH-009-25; **d**. QRB-14-25; **e, f**. *Micropsalliota striata* (CESAR-ATM-009-25, holotype). Scale bars: 15 mm.

**Figure 3.**
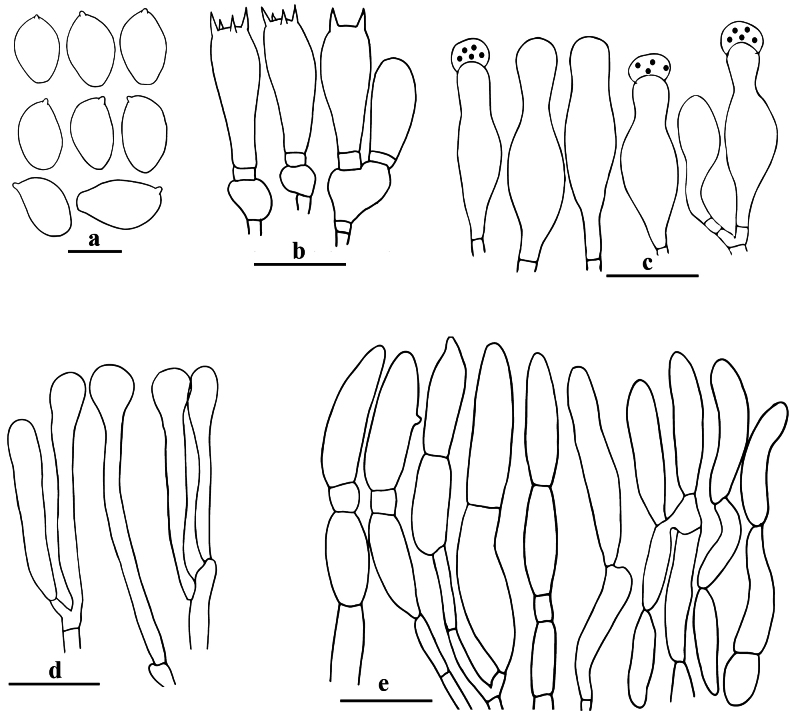
Illustrations of microscopic features of *Micropsalliota latiumbonata* (CESAR-ASH-007-25, holotype). **a**. Basidiospores; **b**. Basidia; **c**. Cheilocystidia (black dots represent the olivaceous content); **d**. Caulocystidia; **e**. Pileus squamules. Scale bar: 5 µm (**a**); 15 µm (**b–e**).

##### Diagnosis.

*Micropsalliota latiumbonata* is a very small-sized species, characterized by a broadly umbonate pileus center, occurring gregariously in a specific habitat of termite mounds, and microscopically with olivaceous capitulum at the apex of cheilocystidia.

##### Holotype. •

Sultanate Of Oman: Dhofar, Salalah, Ashwur, on soil with grasses on termite mounds, 28 August 2025, S. Hussain, Al-Nadabi, A. Al Sidairi, K. Al-Zeidi & A. Al-Raeesi, ASH-007-25 (CESAR-ASH-007-25, holotype), GenBank accession: ITS = PZ127413, 28S = PZ127516, *tef1-α* = PZ386150.

##### Description.

Basidiomata small-sized. Pileus 10–18 mm, at young stage campanulate to hemispherical, becoming convex to plano-convex at mature stage, finally becoming plano-concave to concave in old specimens, with broadly umbonate disc; surface fibrillose-squamulose, fibrils more or less recurved, reddish-brown (10R 5/4–2.5YR 6/4) to dark reddish-brown (10R 3/10–10R 3/14), concentrated at the center, sparser towards the margins; margin appendiculate, whitish; in old specimens the entire basidiomata becoming dark-brown (5YR 1/2–7.5YR 1/2). Context membranous, whitish. Lamellae free, pale pinkish (10R 9/2–7.5YR 9/2) to dark grayish-brown (2.5YR 1/4–7.5R 2/4), broad to ventricose, with fimbriate edge, close with 0–3 series of lamellulae. Stipe 15–30 × 2–3 mm, central, cylindrical, slender, white, surface pruinose to furfuraceous downwards. Context of stipe secreting brownish red (5R 3/10–7.5R 3/10) liquid in some specimens on cutting. Annulus cortinate, whitish.

Basidiospores [141/2/2] (4.5)6.0–7.0(7.8) × (3.5)4.0–4.5(4.7) µm, on average 6.5 × 4.2 µm, Q = 1.4–1.7, av. Q = 1.6, in face view broadly fusiform with a truncate apex, in side view oblong to amygdaliform with slightly flattened on one side, pale brownish, slightly thick-walled, with a prominent apiculus, without germ-pore. Basidia 18–23 × 7.0–8.0 µm, clavate, hyaline, 4-spored, rarely 2-spored. Pleurocystidia absent. Cheilocystidia 32–46 × 9.0–12 µm, hyaline, narrowly utriform to tibiiform capitate apex, capitulum 5–8 µm in diam, smooth, with olivaceous content. Pileus squamules is a cutis, consisting of hyphae 11–15 µm in diam, pale brownish, smooth, cylindrical to elongated, constricted at septa, rarely branched. Caulocystidia 46–88 × 8.0–12 µm, tibiiform to cylindrical, with capitate apex, hyaline. Clamp connection absent in all tissues.

##### Habit, habitat and distribution.

Gregarious or scattered in small groups, saprotrophic, on the soil of a termite mound. Currently only known from southern Oman.

##### Additional specimens examined.

• Sultanate Of Oman: Dhofar, Salalah, Qashrub, on soil under herbaceous plants, 27 August 2025, S. Hussain, Al-Nadabi, K. Al-Zeidi & A. Al-Raeesi, QRB-14-25 (paratype CESAR-QRB-14-25), GenBank accession: ITS = PZ127411, 28S = PZ127514, *tef1-α* = PZ390627; Salalah, Ashwur, on soil with grasses on termite mounds, 28 August 2025, S. Hussain, Al-Nadabi, A. Al Sidairi, K. Al-Zeidi & A. Al-Raeesi, ASH-005-25 (paratype CESAR-ASH-005-25), GenBank accession: ITS = PZ127410, 28S = PZ127513, *tef1-α* = PZ390625; same area and same date, S. Hussain, Al-Nadabi, A. Al Sidairi, K. Al-Zeidi & A. Al-Raeesi, ASH-006-25 (paratype CESAR-ASH-006-25), GenBank accession: ITS = PZ127412, 28S = PZ127515, *tef1-α* = PZ390626; same area and same date, S. Hussain, Al-Nadabi, A. Al Sidairi, K. Al-Zeidi & A. Al-Raeesi, ASH-009-25 (paratype CESAR-ASH-009-25), GenBank accession: ITS = PZ127414, 28S = PZ127517, *tef1-α* = PZ176529.

##### Note.

The new species *Micropsalliota latiumbonata* can be recognized by its growing habit, which mainly fruits on a termite mound; pileus with a broadly umbonate center, covered with reddish-brown squamules; and capitate cheilocystidia, with the capitulum containing olivaceous material. There are several species in the genus with a similar combination of characteristics, such as *M. albosericea* Heinem. & Leelav., *M. ferruginea* T. Gao & Z.W. Ge, *M. fimbriata* T. Gao & Z.W. Ge, *M. gracilis* Heinem., *M. longicystis* T. Gao & Z.W. Ge, *M. nana* T. Gao, H. Qu & Z.W. Ge. *Micropsalliota albosericea* has a whitish fibrillose pileus, basidiospores ovoid or cymbiform with apical thickening and smaller (5–5 × 2.8–3.2 µm), utriform cheilocystidia with capitate apex and hyaline (Heinemann and Leelavathy 1991). *Micropsalliota ferruginea* was recently reported from China, with a larger pileus (35–50 mm diam), covered with recurved squamules, without a prominent umbo, and smaller cheilocystidia (15–33 × 3–10 µm) with hyaline capitulum (Gao et al. 2024). Similarly, *M. gracilis* with an umbonate pileus center, covered with brownish-red squamules, basidiospores amygdaliform to ellipsoid and smaller (5.5–6.5 × 3.0–4.0 µm), ventricose to irregularly tibiiform cheilocystidia with hyaline capitate apex (Zhao et al. 2010). *Micropsalliota longicystis* shares capitate caulo- and cheilocystidia with the new species; however, it is differentiated from *M. latiumbonata* by its smaller basidiospores (5.0–6.0 × 3.0–3.5 µm), hyaline capitulum of cheilocystidia, and the presence of pleurocystidia (Gao et al. 2024). *Micropsalliota nana* differentiated from the new species by its silky fibrillose pileus with obtuse umbo, persistent annulus, basidiospores smaller (4.5–6.0 × 3.0–4.0 µm) ellipsoid to amygdaliform, hyaline capitulum of cheilocystidia, with encrusted hyphae in pileus squamules (Gao et al. 2024).

Based on multigene phylogeny, the close relatives of *Micropsalliota latiumbonata* are *M. appendiculata* D.D. Ivanova & O.V. Morozova, *M. megaspora* R.L. Zhao, Desjardin, Soytong & K.D. Hyde, *M. repanda* Heinem., *M. jiangxiensis* X.H. Ji & G. He, *M. ferruginea*, and the proposed new species *M. striata* sp. nov. *Micropsalliota appendiculata* has been reported from China and Vietnam under broad-leaved trees, with pyriform to subglobose cheilocystidia, presence of pleurocystidia (broadly clavate to clavate-capitate), with encrusted hyphae of pileus squamules (Gao et al. 2024). *Micropsalliota megaspora* can be differentiated from the new species by its larger basidiospores (6–8 × 3.8–4.5 µm), ventricose cheilocystidia with hyaline, subcapitate apices, and incrusted hyphae in pileus squamules (Zhao et al. 2010). Basidiomata of *Micropsalliota repanda* are comparatively larger (pileus diam up to 45 mm), with purplish-red pileal squamules, capitate hyaline (Heinemann 1980). *Micropsalliota jiangxiensis* with pileus 25–32 mm diam, covered with wood-brown fibrils, basidiospores 5.3–6.7 × 2.9–3.4 µm, ellipsoid, cheilocystidia cylindrical to subclavate with capitate apex, hyaline (Ji and He 2023).

Nucleotide differences in the ITS region of both the new species (*Micropsalliota latiumbonata, M. striata*) with their close relative *M. appendiculata* is given in Table 2.

**Table 2.** Nucleotide differences in the ITS sequences of the new species and with their closely similar species, - means deletion.

**Species**	**Collection**	**Variable positions**																		
		**73**	**103**	**189**	**193**	**208**	**220**	**229**	**268**	**281**	**445**	**478**	**502**	**590**	**605**	**612**	**622**	**628**	**629**	**639**
*M. latiumbonata*	ASH-005-25	C	T	A	G	G	A	T	T	A	T	T	A	G	T	A	T	T	C	G
*M. latiumbonata*	ASH-006-25	C	T	A	G	G	A	T	T	A	T	T	G	G	T	A	T	T	C	G
*M. latiumbonata*	ASH-007-25	C	T	A	G	G	A	T	T	A	T	T	G	G	T	A	T	T	C	G
*M. latiumbonata*	ASH-009-25	C	T	A	G	G	A	T	T	A	T	T	G	G	T	A	T	T	C	G
*M. latiumbonata*	QRB-14-25	C	T	A	G	G	A	T	T	A	T	T	G	G	T	T	T	T	C	G
*M. striata*	ATM-009-25	T	G	G	T	A	A	C	G	T	A	G	G	A	T	A	C	T	T	-
*M. appendiculata*	LE F-315913	T	G	G	T	A	T	C	G	T	A	G	G	A	T	A	T	A	-	T
*M. appendiculata*	HKAS131127	T	G	G	T	A	T	C	G	T	A	G	G	A	T	A	T	A	-	T

#### Micropsalliota striata

Taxon classification

Fungi

AgaricalesAgaricaceae

S. Hussain, Al-Nadabi & Al‐Sadi sp. nov.

3039901B-ED86-5E12-BF24-42CCFD9BD4C2

862916

Figs 2, 4

##### Etymology.

The specific epithet ‘striata’ refers to the fine striate pileus margin of the new species.

**Figure 4.**
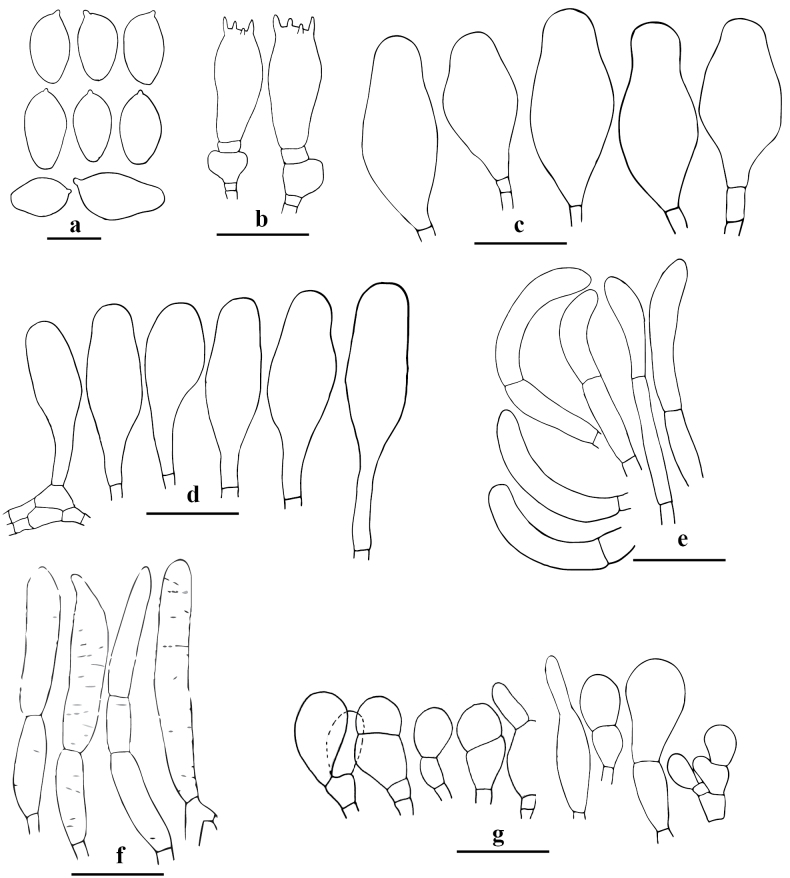
Illustrations of microscopic features of *Micropsalliota striata* (CESAR-ATM-009-25, holotype). **a**. Basidiospores; **b**. Basidia; **c**. Cheilocystidia; **d**. Pleurocystidia; **e**. Caulocystidia; **f**. Pileus squamules (back dashes showing the encrusted walls); **g**. Pileocystidia. Scale bar: 5 µm (**a**); 15 µm (**b–g**).

##### Diagnosis.

*Micropsalliota striata* is a small-sized species characterized by a striate pileus margin, a papillate disc, microscopically with the presence of pileocystidia and caulocystidia.

##### Holotype.

• Sultanate Of Oman: Dhofar, Salalah, Ain Athum, on moist soil with mosses, 25 August 2025, S. Hussain, Al-Nadabi, A. Al Sidairi, K. Al-Zeidi & A. Al-Raeesi, ATM-009-25 (CESAR-ATM-009-25, holotype), GenBank accession: ITS = PZ127415, 28S = PZ127518.

##### Description.

Basidiomata small-sized. Pileus 15–20 mm, at young stage paraboloid to hemispherical, becoming convex to plano-convex and applanate at mature stage, with acute papillate disc; surface fibrillose, fibrils appressed, sparse towards the margin, light-pinkish (2.5R 9/4–10RP 9/4) to light yellowish-pink (7.5R 9/4–7.5R 9/6), concentrated at the disc, light-brown (5YR 4/4–7.5YR 4/4) to reddish-brown (10R 5/4–2.5YR 6/4); margins finely striate, exceeding the lamellae, appendiculate, whitish. Context membranous, whitish. Lamellae free, pale pinkish (10R 9/2–7.5YR 9/2) to light reddish-brown (2.5YR 5/4–2.5YR 6/4), ventricose, with fimbriate edge, close with 1–3 series of lamellulae. Stipe 25–40 × 2–3 mm, central, equal, slightly flexuous, slender, pale-pinkish (2.5YR 9/2–7.5R 9/2) to pale-brownish (10R 5/4–10R 6/4), surface pruinose to pulverulent downwards, glabrous upwards. Context of stipe unchanging on bruising or cutting. Annulus absent.

Basidiospores [104/2/1] (6.2)6.5–7.5(8.3) × (3.5)3.7–4.1(4.5) µm, on average 7.0 × 3.9 µm, Q = 1.6–1.8, av. Q = 1.7, in face view oblong to broadly fusiform with truncate apex, in side view amygdaliform to ellipsoid with slightly flattened on one side, pale brownish, slightly thick-walled, with prominent apiculus, without germ-pore. Basidia 14–17 × 5.5–7.0 µm, clavate to cylindrical, hyaline, regularly 4-spored. Pleurocystidia 28–36 × 9.5–11.5 µm, oblong to clavate or narrowly utriform to cylindrical, abundant, hyaline. Cheilocystidia 23–30 × 8.5–11.5 µm, narrowly utriform to utriform without a capitate apex, hyaline. Pileus squamules is a cutis, consisting of encrusted hyphae, 10–25 µm in diam, pale brownish, cylindrical to elongated, constricted at septa, rarely branched. Pileipellis is a hymeniderm to epithelium, consisting of subglobose to clavate pileocystidia, measuring 11.5–20.5 × 7.0–10.5 µm, hyaline, smooth. Caulocystidia 40–51 × 8.5–10 µm, clavate to cylindrical or cylindro-clavate, without a capitate apex, hyaline. Clamp connection absent in all tissues.

##### Habit, habitat and distribution.

Gregarious or scattered in small groups, saprotrophic, on moist soil with mosses. Currently only known from southern Oman.

##### Note.

The new species *Micropsalliota striata* is characterized by its acute papillate pileus disc, with striate pileus margins, having clavate to cylindrical caulocystidia, narrowly utriform to utriform pleuro- and cheilocystidia without a capitate apex, and comparatively larger basidiospores (6.5–7.5 × 3.7–4.1 µm) than *M. latiumbonata*. In the genus, there are several species with similar kind of characteristics, like: *M. appendiculata*, *M. gigaspora* T. Gao & Z.W. Ge, *M. pileocystidiata* P.B. Patil & S.A. Vaidya, *M. pleurocystidiata* Heinem. & Little Flower, *M. pseudoarginea* Heinem. and *M. xanthorubescens* Heinem. *Micropsalliota appendiculata* can be differentiated from the new species by its pyriform to subglobose cheilocystidia measuring (16–25 × 10–13 µm), pleurocystidia broadly clavate to clavate-capitate, pileipellis without pileocysidia (Gao et al. 2024). *Micropsalliota gigaspora* with a subumbonate to applanate pileus disc with crenate margins, stipe with a persistent annulus, lengthy basidiospores (7–9 × 4–5.5 µm), broadly-clavate to clavate cheilocystidia, and 2- and 4-spored basidia (Gao et al. 2024). *Micropsalliota pileocystidiata*, only known from the type location (Maharashtra, India), is a medium- to large-sized species with pyriform pileocystidia (Patil et al. 2022). *Micropsalliota pleurocystidiata* is a large-sized species, with a broadly umbonate pileus disc, basidiospores amygdaliform and smaller (5.0–6.0 × 3.0–4.0 µm), broadly ventricose to subcylindrical cheilocystidia (Zhao et al. 2010). *Micropsalliota pseudoarginea* with glabrous to finely pruinose cap surface, smaller basidiospores (4.0–5.0 × 2.5–3.2 µm), ellipsoid to subcymbiform shape, with clavate to ventricose cheilocystidia (Zhao et al. 2010). *Micropsalliota xanthorubescens* is a medium-sized species with a subumbonate pileus disc, clavate to ventricose cheilocystidia (Zhao et al. 2010).

Both the newly proposed species, *Micropsalliota striata* and *M. latiumbonata*, can be differentiated by several characteristics. Pileus disc in the former is acute papillate as compared to the broadly umbonate disc in the latter; the cap surface is sparsely covered with pinkish, appressed fibrils in *M. striata*, in contrast to much denser, recurved, and brownish fibrils in *M. latiumbonata*. Pleurocystidia and pileocystidia are completely absent in *M. latiumbonata*. Cheilocystidia in *M. striata* are without a prominent capitate apex; however, the cheilocystidia of *M. latiumbonata* have a characteristic capitulum with olivaceous content.

## Discussion

The present study contributes to the description of two new species of *Micropsalliota* from Oman, a region that remains underexplored for agaric diversity. In the phylogenetic analyses (Fig. 1), both the proposed new species were grouped in the Clade megaspora. Species in this clade have small pilei with fibrillose-squamulose surface, fibrils/squamules usually brownish, concentrated at the center, the center more or less umbonate, basidiospores ellipsoid to amygdaliform with apical thickening, cheilocystidia ventricose to clavate or subglobse with capitate or subcapitate apex. To the best of our knowledge, *M. latiumbonata* is the only species having capitate cheilocystidia with olivaceous content in the capitulum.

In this study, species of *Micropsalliota* were recovered in eleven major clades, similar to a previous phylogeny of the genus (Gao et al. 2024). A couple of species *M. bispora* J.Q. Yan, S.Nan Wang & H. Zeng and *M. vulgaris* J.Q. Yan, S.N. Wang & H. Zeng, were described after Gao et al. (2024), however, these species were not placed in any known clade of the genus (Yan et al. 2025). In the current phylogeny, *M. bispora* was grouped in Clade furfuraceae. Species in this clade have larger basidiospores size about 6.9 × 3.8 µm on average (Zhao et al. 2010; Gao et al. 2024). Similarly, *M. vulgaris* recovered in Clade cortinata. Species in this clade have a cortinate partial veil remnants at the pileus margin (Zhao et al. 2010; Gao et al. 2024). Clade bifida being the largest clade in *Micropsalliota* in terms of species, with the inclusion of *M. pakistanica* Maula, Saba, Asif & Akram (Maula et al. 2025), the number of species increased to 26. Most of the species in Clade bifida have small-sized basidiomata, except *M. brunneosquamata* Linda J. Chen, R.L. Zhao & K.D. Hyde and *M. geesterani* with large basidiomata and thickened fleshy pileus (Gao et al. 2024). One species was recently published with valid description; however, the name was invalid (as *Micropsalliota rosea* Akram, Maula, Saba & Asif (Maula et al. 2025). The species was redescribed with valid name *Micropsalliota rosea* Akram, Maula, Saba & Asif, but not assigned to any clade of the genus (Javeed et al. 2025). In the present study, *M. rosea* was grouped in Clade lateritia.

Species of *Micropsalliota* are saprotrophs in tropical habitats, usually fruiting on humus-rich degrading soil, under forest trees or roadsides (Zhao et al. 2010). In the present study, four collections of one of the new species *Micropsalliota latiumbonata* (ASH-005-25, ASH-006-25, ASH-007-25 and ASH-009-25) were found on termite mound, suggesting a possible ecological association between these mushrooms and termites. To the best of our knowledge, no species in *Micropsalliota* has been found on any termite mounds. However, four species of *Hymenagaricus* were recently reported from Dhofar region, Oman, occurring on termite mounds (Hussain et al. 2024b, 2026). Beside the termite-associated mushroom genus *Termitomyces*, a number of other mushroom genera, such as *Agaricus*, *Lepiota*, *Leucocoprinus*, *Xanthagaricus*, *Conocybe*, and *Clitopilus* were encountered fruiting on termite mounds in Dhofar region (person. Comm.). Termite mounds that contain abundant moisture and nutrients may provide a suitable habitat for mushroom growth and development. Consequently, further research is necessary to investigate the association between mushrooms and termite mounds in greater detail.

## Supplementary Material

XML Treatment for Micropsalliota latiumbonata

XML Treatment for Micropsalliota striata

## Additional information

### Artificial Intelligence (AI) use

The authors accept full responsibility for the content of the manuscript, including the disclosure of any use of AI.

No AI tools were used in the preparation of this manuscript.

### Author ORCIDs

S. Hussain https://orcid.org/0000-0002-5772-7206

A. Al Sidairi https://orcid.org/0009-0008-5476-2621

A.M. Al-Sadi https://orcid.org/0000-0002-3419-8268

### Data availability

All sequence data generated in this study (Table 1) are available in NCBI GenBank (https://www.ncbi.nlm.nih.gov/genbank/). The multigene dataset used for phylogenetic analyses is available at Figshare (https://doi.org/10.6084/m9.figshare.32259831).
